# The impact of smoking on periodontal status and dental caries

**DOI:** 10.18332/tid/152112

**Published:** 2022-08-29

**Authors:** Arzu Beklen, Nichal Sali, M. Burak Yavuz

**Affiliations:** 1 Department of Periodontology, Faculty of Dentistry, Eskisehir Osmangazi University, Eskisehir, Turkey

**Keywords:** periodontal disease, caries, DMFT, smoking, oral health

## Abstract

**INTRODUCTION:**

Investigations to explore the relationship between smoking and its oral manifestations are important to clinicians. Among these oral manifestations, periodontal diseases and dental caries have still a controversial association. This study aims to analyze the effect of smoking on periodontal disease and caries and their relevance to each other.

**METHODS:**

Data on demographic and clinical features were retrieved from 7028 patients. Smoking status was categorized as a smoker, non-smoker, former smoker and passive smoker. Each patient received a diagnosis according to the new classification system for periodontal disease, in which periodontal disease is divides into stages (PS). The carries status was diagnosed by evaluating the decayed, missing, and filled teeth (DMFT) index.

**RESULTS:**

Of the patients, 66.6% were non-smoker women, whereas 53.7 % of passive smokers were women. Being a worker and having a Bachelor’s degree was associated with a higher likelihood of getting diagnosed with periodontal disease and caries in smokers. Smoking significantly influences periodontal disease severity and DMFT values (p<0.001). This becomes more evident in former smokers by showing the highest severe periodontal problems (PS3: 29.7% and PS4: 18.9%), and the highest DMFT mean (16.4 ± 7.4) Accordingly, persons having high DMFT had significantly the most severe periodontal disease, namely PS4 (p<0.05).

**CONCLUSIONS:**

Smoking is associated with higher caries prevalence and more severe periodontal disease, and DMFT tend to increase with the severity of periodontitis in the same subjects.

## INTRODUCTION

Tobacco smoking is one of the most prevalent public health problems negatively influencing systemic and oral health problems, such as periodontal diseases and dental caries^1^. Periodontal disease comprises a wide range of inflammatory conditions affecting the teeth-supporting structures, resulting in tooth loss. The disease can affect up to 50% of the worldwide population, which is expected to increase in the coming years due to growth in the ageing population^2^. Likewise, dental caries, otherwise known as tooth decay, is also one of the most prevalent oral diseases that affects individuals throughout their lifetime^3^. Despite clear evidence of smoking on periodontal disease^4^, there is a lack of information on dental caries and particularly co-occurrence of both diseases linked with smoking^5^. The effect of smoking on the progression of periodontal diseases has been firmly accepted, and the underlying mechanisms have been investigated extensively^4^. However, the impact of tobacco on dental caries, another problem that seriously affects societies, has not been considered to this extent. Furthermore, the studies covering the periodontal disease/caries/smoking triangle have been neglected. Few studies have evaluated the relationship between periodontal disease and caries, in which contradictory results were obtained^6-8^. Some studies showed no association between periodontal disease and caries^8^, some showed a negative relationship^7^, and some indicated that caries risk increased with the severity of periodontal disease^6^. Although studies show different relationships, there are logical explanations for the acceptance of smoking as a common risk factor in the relationship between periodontal disease and caries. Smoking, in the simplest term, affects the oral environment’s temperature and humidity and negatively affects the buffering capacity of saliva^9^. This altered living environment disrupts the average healthy balance of oral bacteria and causes caries bacteria to dominate^10,11^. Likewise, toxic products such as nicotine in cigarettes will affect the immune response in the surrounding tissues and cause periodontal disease^10^.

In 2017, the classification of periodontal diseases was changed. The new periodontal classification system has a staging system, which intends to classify the severity and extent of a condition based on the measurable amount of destroyed and/or damaged tissue^4^. Because smoking is a significant common variable for both diseases, shifting the research on the relation between periodontal disease stages and caries co-occurrence is even more critical for smokers by defining the new terminology. In 2017, the consensus report in the joint EFP/ORCA workshop concluded the need for future research to improve the understanding of smoking as a risk factor for the simultaneous occurrence of caries and periodontal diseases^12^.

Because early evidence has indicated that smokers are highly susceptible to periodontal disease and caries^1^, identifying sociodemographic factors, habits and disease prevalence also becomes crucial to implementing new strategies when managing periodontal diseases and dental caries^13,14^ in smoking patients. The present study aimed to test the hypothesis that there is an association between periodontal diseases and dental caries, sharing smoking as a risk factor in the same patients.

## METHODS

### Study population

This cross-sectional study was conducted at Eskisehir Osmangazi University, in the Department of Periodontology, through analyzing patient files diagnosed with periodontal disease between 2015 and 2020. The patients were referred to the periodontology clinic due to the prior clinical and radiographic examinations at the department of oral and maxillofacial radiology. The patient files, all of which were filled in completely, were included in this study (N=7028). Under specializing dentists’ supervision, dental trainees performed complete periodontal examinations on each individual. Caries information was acquired by analyzing the radiographs and keeping the records assessed visually with a mirror and probe at the referring clinic. Data on sociodemographic details, oral health behavior and smoking habit were obtained through interviews. The Eskisehir Osmangazi University Ethics Committee approved the study (Ethical permit: 2021-52).

### Periodontal parameters

The patients’ clinical periodontal parameters such as bleeding on probing, pocket depth, clinical attachment level and dental radiographs were assessed to determine the periodontal health status. Periodontal disease status was categorized according to the new periodontal disease classification accepted in the 2017 world workshop, which allows the differentiation between the different types of periodontal disease, briefly periodontal health, gingivitis or periodontitis, from mild to the severe form. Because dental plaque-induced gingivitis (G) may arise on an intact periodontium (IP) or a reduced periodontium (RP), we categorized our gingivitis patients as GIP or GRP, respectively. An intact periodontium refers to an absence of detectable periodontal tissue loss. In contrast, a reduced periodontium refers to the periodontium with pre-existing loss of periodontal tissue in a stable situation. According to the classification of the Academy of Periodontology and the European Federation of Periodontology, when interdental clinical attachment loss (CAL) was found at ≥2 non-adjacent teeth, or buccal or lingual CAL ≥3 mm with >3 mm pocket depth detectable at ≥2 teeth, the patient was considered as periodontitis case^4^. In the periodontitis group considering different variables such as bone loss amount or type of bone defects, the severity and the extent of the disease were differentiated into stages (stages I through IV), in which stage IV represents the most severe patients. Stage I presents 1–2 mm CAL, and stage II offers 3–4 mm CAL. Stage 3 and stage 4 depend on missing teeth. Both stages III and IV present CAL ≥5 mm, whereas stage 3 has ≤4 tooth loss and stage 4 has ≤5 or more tooth loss.

### Caries findings

DMFT (sum of decayed, missed, and filled teeth) index^15^ was used to express the caries prevalence numerically. Based on data regarding several degraded (D), missing (M) and filled (F) teeth (T), the mean DMFT index was determined. The teeth with a cavity were marked under the D category. Filled and crowned ones were marked under the F category, and missing teeth were marked under the M category.

### Smoking

The standard National Health Interview Survey of U.S. Public Health Service (NHIS)16 current smoking definitions, which screens for lifetime smoking ≥100 cigarettes were used; smokers were divided into four groups: 1) Current smoker (who has smoked 100 cigarettes in their lifetime and who currently smokes cigarettes); 2) Non-smoker (who has never smoked, or has smoked less than 100 cigarettes in their lifetime); 3) Former smoker (who smoked at least 100 cigarettes in their lifetime but quit smoking at the time of dental examination); and 4) Passive smoker (who inhaled secondhand smoke).

### Statistical analysis

The collected data were recorded in MS Excel 2003 and exported to SPSS Statistical Software version 20.0 (Armonk, NY: IBM Corp.). Data are presented as frequencies (n), mean ± standard deviation (SD), or percentages unless specified otherwise. Descriptive statistics were generated on patient sociodemographic and all other variables. The averages of the measurements in different parameter groups were examined with a t-test or one-way ANOVA. If there was a significant difference between the groups, the Tukey post-test was followed to determine from which group the difference originated. The relationship between smoking status, diagnosis groups, and other parameters was analyzed by chi-squared tests. The results were assessed at a 95% confidence interval and a significance level of 0.05.

## RESULTS

Table 1 shows the results of demographic characteristics of the patients regarding smoking status. Accordingly, smoking status differs significantly according to age group, gender, education level, occupation and oral health maintenance (p<0.05). Most non-smokers (66.6%) were women, whereas 67.6% of men reported themselves as former smokers. The most evident differences were observed between those aged 18–34 years and those aged between 45–64 years. The youngest persons (aged 18–34 years) made up 58.7% of the smoker group and 45.3% of the passive smoker group. Between the ages of 45–60 years, 40% of the group stopped smoking. In the occupation category, the workers were likelier to have oral health problems in all smoking groups, while those in the non-smoker group most frequently (31.5%) were students. Regarding education, the patients who graduated with a Bachelor’s degree (39.7%) and high school (35.1%) smoked mostly. Whereas most persons in the group of non-smokers (43.6%), former smokers (59.5%) and passive smokers (45.3%) belonged to those with an education level of Bachelor’s degree. Most of the patients brushed their teeth twice daily in all smoking groups. In contrast, over 80% of patients in all smoking groups reported themselves as not dental-flossing.

**Table 1:** Demographic characteristics of smokers and non-smokers (N=7028)

*Characteristics*		*Smoking status*								*p^a^*
		*Smoker*		*Non-smoker*		*Former smoker*		*Passive smoker*	
		*n*	*%*	*n*	*%*	*n*	*%*	*n*	*%*
**Sex**	Male	1108	52.8	1558	33.4	50	67.6	88	46.3	0.000*
	Female	992	47.2	3106	66.6	24	32.4	102	53.7	
**Age** (years)	18–34	1232	58.7	2600	55.7	22	29.7	86	45.3	0.000*
	35–44	492	23.5	868	18.6	20	27.0	54	28.4	
	45–64	360	17.2	1048	22.5	30	40.5	50	26.3	
	65–74	14	0.7	138	3.0	2	2.7	0	0.0	
	≥75	0	0.0	12	0.3	0	0.0	0	0.0	
**Occupation**	Student	554	26.4	1468	31.5	6	8.1	46	24.2	0.000*
	Worker	1008	48.0	1488	31.9	46	62.2	90	47.4	
	Unemployed	442	21.0	1338	28.7	12	16.2	50	26.3	
	Retired	96	4.6	370	7.9	10	13.5	4	2.1	
**Education level**	Primary	194	9.2	554	11.9	6	8.1	20	10.5	0.000*
	Middle school	154	7.3	370	7.9	2	2.7	10	5.3	
	High school	738	35.1	1298	27.8	18	24.3	62	32.6	
	Vocational school	140	6.7	248	5.3	4	5.4	8	4.2	
	University	834	39.7	2034	43.6	44	59.5	86	45.3	
	Postgraduate	40	1.9	160	3.4	0	0.0	4	2.1	
**Toothbrushing frequency**	Does not brush	56	2.7	110	2.4	0	0.0	4	2.1	0.000*
	Sometimes	336	16.0	602	12.9	18	24.3	38	20.0	
	Once/day	682	32.5	1364	29.2	26	35.1	52	27.4	
	Twice/day	890	42.4	2296	49.2	30	40.5	74	38.9	
	Three times/day	136	6.5	292	6.3	0	0.0	22	11.6	
**Flossing**	Yes	274	13.0	848	18.2	10	13.5	24	12.6	0.000*
	No	1826	87.0	3816	81.8	64	86.5	166	87.4	

a Chi-squared test.

Six different disease forms were recorded regarding the periodontal status of the patients, from mild to more severe conditions. The clinical gingival health on an intact periodontium (GIP) and clinical gingival health on a reduced periodontium (GRP) are at baseline, whereas the remaining more severe forms range from PS1 to PS4. Table 2 shows that the smoking status differs significantly according to periodontal disease severity (p<0.05). In the smoker (32.8%), non-smoker (37.5%) and passive smoker (28.4%) groups, the majority of the periodontal health status was recorded at the GIP level. In contrast, in the group of former smokers, the disease severity for PS3 in 29.7%. The second highest disease distribution rates were recorded at the PS1 level (19.8%) in smokers, at PS1 (17.8%) in non-smokers, at GIP (21.6%) in former smokers and PS1 (16.8%) in passive smokers. The most severe PS4 was the highest (18.9%) in former smokers. Notably, the frequencies show that former smokers had the highest percentage of PS3, followed by passive smokers. The difference between smokers and non-smokers for PS3 was less than 1%. Similarly, in PS4, the former smokers had the highest value, followed by passive and current smokers.

**Table 2:** Periodontal diagnosis according to smoking status (N=7028)

*Periodontal disease severity*	*Smoking status*							
	*Smoker*		*Non-smoker*		*Former smoker*		*Passive smoker*	
	*n*	*%*	*n*	*%*	*n*	*%*	*n*	*%*
GIP	688	32.8	1748	37.5	16	21.6	54	28.4
GRP	206	9.8	556	11.9	4	5.4	18	9.5
PS1	416	19.8	828	17.8	8	10.8	32	16.8
PS2	330	15.7	776	16.6	10	13.5	30	15.8
PS3	254	12.1	526	11.3	22	29.7	28	14.7
PS4	206	9.8	230	4.9	14	18.9	28	14.7

GIP: clinical gingival health on an intact periodontium. GRP: clinical gingival health on a reduced periodontium. PS: periodontitis stage. Chi-squared test, p=0.000 (Pearson’s R=0.021, Spearman correlation=0.033).

Results of the relationship between smoking status and DMFT measurement were examined; it was observed that the measurements differed significantly according to the smoking status (p<0.05) (Table 3). The Tukey test revealed that: 1) The mean of smokers is significantly higher than non-smokers, former and social smokers for D measurement. In addition, the mean of social smokers is significantly higher than that of non-smokers and former smokers; 2) The mean of former and passive smokers is significantly higher than that of smokers and non-smokers for M measurement; and 3) The mean of former smokers was significantly greater than that of smokers and non-smokers for F measurement. In overall, the mean DMFT of former (16.4 ± 7.4) and passive smokers (15.5 ± 6.5) was significantly higher than that of smokers (12.9 ± 6.4) and non-smokers (11.6 ± 6.7).

**Table 3:** Decayed, missing and filled teeth by smoking status (N=7028)

*Smoking status*			*Decayed*		*Missing*		*Filled*		*DMFT*	
	*n*	*%*	*Mean (SD)*	*p*	*Mean (SD)*	*p*	*Mean (SD)*	*p*	*Mean (SD)*	*p*
Smoker	2100	29.9	4.8 (2.7)		4.2 (4.5)		3.9 (3.9)		12.9 (6.4)	
Non-smoker	4664	66.4	3.7 (2.5)	0.000*	4.1 (4.8)	0.000*	3.8 (3.9)	0.017*	11.6 (6.7)	0.000*
Former smoker	74	1.1	3.5 (2.2)		7.8 (6.3)		5.1 (4.2)		16.4 (7.4)	
Passive smoker	190	2.7	4.3 (2.8)		6.8 (6.3)		4.3 (4.0)		15.5 (6.5)	

Finally, we compared the means of DMFT measurements with periodontal disease severity for smokers. As shown in Table 4, DMFT differs significantly according to the periodontal diagnosis (p<0.05). The DMFT value tended to increase as the severity of periodontal disease increased. The highest DMFT was detected in PS4 (19.5 ± 7.3), whereas the lowest DMFT was recorded at the GIP level (8.5 ± 4.7). When the sub-groups (e.g. PS1 vs PS4) are compared, DMFT differs significantly between different stages of periodontal diseases in all parameters, though the difference decreases in the decayed tooth values.

**Table 4:** Periodontal status by decayed, missing and filled teeth (N=2364)

	*n*	*%*	*Decayed*			*Missing*			*Filled*			*DMFT*		
			*Mean (SD)*	*Versus*	*P*	*Mean (SD)*	*Versus*	*P*	*Mean (SD)*	*Versus*	*p*	*Mean (SD)*	*Versus*	*p*
**GIP**	758	32.1	3.8 (2.4)	GRP	0.006	1.6 (2.0)	GRP	0.000	1.6 (2.0)	GRP	0.000	1.6 (2.0)	GRP	0.000
				PS1	0.000		PS1	0.000		PS1	0.000		PS1	0.000
				PS2	0.000		PS2	0.000		PS2	0.000		PS2	0.000
				PS3	0.000		PS3	0.000		PS3	0.000		PS3	0.000
				PS4	0.000		PS4	0.000		PS4	0.004		PS4	0.000
**GRP**	228	9.6	4.1 (2.8)	GIP	0.006	2.6 (3.1)	GIP	0.000	2.6 (3.1)	GIP	0.000	2.6 (3.1)	GIP	0.000
				PS1	0.204		PS1	0.000		PS1	0.519		PS1	0.000
				PS2	0.145		PS2	0.000		PS2	0.000		PS2	0.000
				PS3	0.236		PS3	0.000		PS3	0.181		PS3	0.000
				PS4	0.029		PS4	0.000		PS4	0.219		PS4	0.000
**PS1**	456	19.3	4.2 (2.7)	GIP	0.000	3.8 (3.6)	GIP	0.000	3.8 (3.6)	GIP	0.000	3.8 (3.6)	GIP	0.000
				GRP	0.204		GRP	0.000		GRP	0.519		GRP	0.000
				PS2	0.805		PS2	0.000		PS2	0.000		PS2	0.000
				PS3	0.973		PS3	0.000		PS3	0.401		PS3	0.000
				PS4	0.195		PS4	0.000		PS4	0.061		PS4	0.000
**PS2**	370	15.6	4.2 (2.6)	GIP	0.000	6.4 (4.7)	GIP	0.000	6.4 (4.7)	GIP	0.000	6.4 (4.7)	GIP	0.000
				GRP	0.145		GRP	0.000		GRP	0.000		GRP	0.000
				PS1	0.805		PS1	0.000		PS1	0.000		PS1	0.000
				PS3	0.852		PS3	0.000		PS3	0.000		PS3	0.604
				PS4	0.275		PS4	0.000		PS4	0.000		PS4	0.000
**PS3**	304	12.8	4.2 (2.7)	GIP	0.000	7.2 (5.2)	GIP	0.000	7.2 (5.2)	GIP	0.000	7.2 (5.2)	GIP	0.000
				GRP	0.236		GRP	0.000		GRP	0.181		GRP	0.000
				PS1	0.973		PS1	0.000		PS1	0.401		PS1	0.000
				PS2	0.852		PS2	0.000		PS2	0.000		PS2	0.604
				PS4	0.237		PS4	0.000		PS4	0.016		PS4	0.000
**PS4**	248	10.5	4.4 (3.2)	GIP	0.000	11.4 (7.3)	GIP	0.000	11.4 (7.3)	GIP	0.004	11.4 (7.3)	GIP	0.000
				GRP	0.029		GRP	0.000		GRP	0.219		GRP	0.000
				PS1	0.195		PS1	0.000		PS1	0.061		PS1	0.000
				PS2	0.275		PS2	0.000		PS2	0.000		PS2	0.000
				PS3	0.237		PS3	0.000		PS3	0.016		PS3	0.000
**P**				0.000*			0.000*			0.000*			0.000*	

GIP: clinical gingival health on an intact periodontium. GRP: clinical gingival health on a reduced periodontium. PS: periodontitis stage.

## DISCUSSION

This is the first study exploring the relationship between periodontal diseases and caries in smokers using the new identified periodontal classification system. Smoking is a significant public health problem at global and national levels. It contributes to a high rate of dental caries development and a substantial increase in periodontal diseases^4,5^. We categorized smokers into four groups: active, former, non-smoker and passive smoker^17^. We found that the size of the smoker and non-smoker groups were more significant than other groups, which resembled a distribution pattern similar to the data of the Turkish Statistical Institute announced in 2019. The official data in Turkey show that the rate of individuals who smoke was 28.0%, while the rate of individuals who did not smoke (quits and non-smokers) was 68%^18^. In this sense, our groups were compatible with the general distribution of the country.

Comparable data on the prevalence of tobacco consumption among different groups is generally challenging due to the lack of disaggregation by occupation, sex, and age^19^. Nonetheless, the evidence demonstrates that smoking can be four times higher among men than women globally^20^. In this sense, our results are consistent with previous studies, in which smoking prevalence was much higher among men than women. We also found that women are more successful in stopping smoking than men. Despite this, women are more exposed to tobacco around them than men. This tendency was also shown in the report of WHO, in which female non-smokers were more likely to be exposed to secondhand tobacco smoke. This may be explained by the fact that the changing norms in women’s roles, such as being more active in life’s daily demands, put women and the young generation at risk for smoking^14,20^.

In North Europe, the smoking cessation rate increases by the age of 60 years, while in Europe, the young generation mainly intends to stop smoking around their forties^21^. Our results complement reports from North Europe, where we observe the highest success rates after the forties. These results may be explained by business life since business life leads to bidirectional changes in smoking attitude^22^. Although we did not determine the details of working conditions from the previous studies, it could be interpreted that difficulties of working conditions may tend to increase smoking rates. In contrast, a satisfied work life is likely to diminish smoking rates^23,24^. Workplaces have access to a large number of people, and the workplaces are an important setting to increase the number of quitters. Hence, obtaining the highest cessation rates among the working individuals was not a surprising result. Social support from co-workers must be considered a critical factor for many workplace health promotion programs^25^.

The other remarkable finding of the study was that the individuals stated they brushed their teeth twice a day, whether they smoked or not. In the previous work of our group, we found that individuals know the importance of oral health and the necessity of hygiene. On the other hand, even though the patients know that smoking would damage their oral health, they continue to smoke^26^. Now we have a much clearer picture of smokers, in which irregular brushing is more common for ex-smokers and social smokers. Further research is needed to unravel these inconsistent behaviors. Yet, in line with the literature, varying rates are generally expected for behaviors, particularly on smoking and alcohol, which in turn involve changes in rates^27^. Furthermore, in the past years, several changes were made in the country regarding the regulation of tobacco consumption, such as issuing a nationwide smoking ban in public places, which might have indirectly affected the patients’ attitude in reporting their actual habits^28^. The patients who perceive public disapproval of smoking may report lower smoking rates^29^.

Then in the second step, we analyzed the relationship between smoking and disease increments in different smoking groups. We found that smoking and the disease increment depended on the smoking status. Furthermore, independent of the smoking type, the smoker groups presented more severe forms of periodontal disease with the highest risk of bone and tooth loss, namely PS3 and PS4. This agrees with the overwhelming amount of currently available evidence in active smokers^30^. Unlike previous studies on smoking, we conducted more thorough analyses by utilizing a new classification system and assessing former and passive smokers. The remarkable finding in our research is that similar advanced periodontal destruction occurs in those who are former smokers as well as those who are passively affected. We grouped our patients based on their answers and subsequently the number of cigarettes they smoked throughout their lives^31^. Patients might not be correct about numbers. The most accurate would be, of course, the examination of biological evaluations, such as saliva^32^. Nevertheless, we have clearly observed that, those who were under the influence of any type of smoking were more prone to advanced periodontal disease according to the new classification system. Furthermore, PS3 and PS4, which were the most advanced forms of periodontal disease, were recorded at the highest percentages in former smokers followed by passive smokers. Our interpretation is, since the majority of former smokers quit due to the tobacco-related serious health conditions^33^, most likely the periodontal tissues are also affected seriously^34^ as much as other parts of the body. Likewise, the individuals who are exposed to secondhand cigarette smoke show the severe stages of periodontal destruction. It is worth mentioning that, compared to active smokers, passive smokers inhale lower doses of toxins, whereas the secondhand tobacco smoke gives similar toxins to oral tissues as that inhaled by current smokers^35^. In this sense, it would not be difficult to predict that the same destructive mechanism happens on periodontal tissue in passive tobacco smokers as well.

Although secondhand tobacco smoke is not considered a health risk by people who are not smokers^36^, epidemiologic studies of non-smokers exposed to secondhand tobacco smoke show that such exposure causes the same diseases as active smoking^37^. Moreover, apart from the direct effect of smoking on oral health, its cosmetically undesirable effects cause people to pay attention to their oral hygiene practices. A healthy smile is of high importance for adults and most of the people do not want a smile that is discolored or causing bad breath. In this sense, the oral hygiene plans that smokers apply to both reduce the negative effect on their oral health and to eliminate the visible side effects support the periodontal health, which in turn slows down the periodontal destruction compared to passive smokers.

DMFT as an index is the key measure of caries prevalence in dental epidemiology^38^. Our findings on the analysis of the DMFT and the influence of smoking were consistent with our periodontal outcomes. Contrary to some studies, we found higher DMFT values in former and passive smokers than active smokers. When we consider the literature in its settings, the potential effects of active smoking on the biology of caries development focuses on the changes in saliva and the dental plaque^11^. This, however, does not take into consideration other variables, like sugar intake. Whatever the true mechanism of the process, it is always important to remember the well-known limitations of the DMFT index, which might underestimate caries prevalence^39^. Because DMFT is based on clinical evaluation, the clinician might miss some hidden caries. In the comparison of periodontal sub-groups to DMFT index, the results revealed lower correlation between dental caries and periodontal disease stages. Considering that in dental clinics, the filled and the missing teeth records are kept more accurately than decayed teeth, the significant difference found between F/M/T values and the periodontitis stages supplements more the general observations in dental clinical practice. If we compare GIF patients with other forms of periodontal diseases, we see that decayed teeth and the progression of periodontal disease are associated. In other words, GIF patients, who have regular check-ups with a dentist and good oral hygiene at home have less decayed teeth and less severe forms of periodontal disease. In contrast, this correlation is not seen in individuals with more severe periodontal disease, if the decayed teeth are taken as a single parameter. In this situation, the neglected hygiene rules by patients must be interpreted along with the limitations of DMFT, such as access to health insurance^40^, which in turn limits making definite conclusions about smoking and DMFT amounts. In addition to DMFT related limitations, the individual answers on cessation can differ greatly among people as well^41^. Clearly, main biomarkers such as baseline cotinine levels are critical to verify the real cessation status^42^. On the other hand, like in our study, if the biochemical data are not available for participants, it is important to remember that, the definition of former or non-smoker is almost exclusively dependent on behavioral or symptomatic indices^43^. Despite these limitations, this study adds secondhand smoking as a high risk of more caries development.

The analysis of the co-occurrence of dental caries and periodontitis suggests that there is an association between these two diseases in smokers. Definitely the biological plausibility due to the combination of different factors, including social, economic, psychological and other sociodemographic variables, affect the development of both diseases^44,45^. In this sense, the findings cannot be easily compared with earlier studies. However, the results allow one to conclude that a higher total prevalence of clinical gingival health on an intact periodontium is recorded in subjects only with the lowest DMFT scores. Individuals with the lowest number of untreated caries presented a prevalence of the mildest periodontal problem^5^. Conversely, the recorded highest DMFT was in PS4 and the high DMFT value was due to the missing teeth. In this sense, rather than untreated teeth, the missing teeth are the underlying cause of high DMFT in severe forms periodontal disease. These findings highlight the importance of modifiable preventive strategies to be activated in smokers by the oral health team, such as increasing the visits to dentists for treatment and modifying oral hygiene habits^46,47^. However, as suggested by some authors, when analyzing the relationship between two diseases, the need of a method that measures in a multidimensional manner, such as the power of both diseases to trigger each other, must be considered carefully^48^. As such, periodontal attachment loss could lead to the exposure of tooth’s root surface, which in turn increases the risk of caries^49^. In a recent systemic review, the higher rates of root caries were associated with patients with periodontal diseases^50^. Similarly, our findings highlight that the higher number of caries was found at the time when gum recession existed and the roots were exposed, but most teeth were still in the mouth. Contrary, in the more advanced stages, where the bone loss has progressed and teeth were lost, the number of missing teeth differed enormously.

## CONCLUSIONS

The relationship between dental caries and periodontal disease is still controversial. Even though the risk factors of both diseases are similar, there are certain differences in the microbiological and etiopathological points, which make them distinctly different infections. On the contrary, clinical manifestations of both caries and periodontal diseases can be controlled easily through regular removal of plaque and regular toothbrushing. Therefore, increasing knowledge of the role of risk factors, namely smoking, can enable public health policies to be implemented that reduce the probability of progression to pathology. Moreover, it is important to actively adopt disease prevention strategies and multidisciplinary actions to achieve early pathological detection and thereby early treatment.

## Data Availability

The data supporting this research are available from the authors on reasonable request.
